# Discussions of the Ohio State Dental Society

**Published:** 1888-01

**Authors:** 


### Discussions of the Ohio State Dental Society.

### DR. BERRY'S PAPER.

Dr. Harroun, Prof. Taft, and others, disagreed materially with the sentiments of the paper. Nowadays, they said, first-class operators rarely have occasion to extract teeth and the professional extractors do not extract as many teeth that ought to be saved as they formerly did, being deterred by the agitation of the subject in the societies and the periodicals.

Dr. E. G. Betty ; I am astonished that a man of Dr. Berry's standing should put on paper and sign his name to such an outrageous doctrine, i. e., that a good tooth should be extracted if the patient insists upon it, and I want to enter my protest against it. If of no more value than that, why does Dr. Younger take the trouble to implant, or put back, just such teeth? I would not allow any portion of a root to remain. Dr. Harroun told us last year of an easy way of removing them by the use of the engine with suitable burrs. Syncope is failure of the heart resulting in anæmia of the brain. Place the patient in a recumbent position to restore the circulation of the brain—the blood by its own pressure will refill the depleted vessels.

Dr. J. Taft: Syncope occurs very frequently from a mental impression and not solely from physical disturbance. If that is true then it is possible to avert it by our manner and actions toward the patient. Sometimes a patient faints before the operation is begun.

DR. WRIGHT'S PAPER "REFLEX-(IONS)."

Dr. J. Taft: If there is this interdependence between the different parts of the body, should not all the specialists confer together—the laryngologists, the opthalmologists, etc.

Dr. H. A. Smith: I think the discussions brought about by the *Medical Record* articles have resulted in good to both professions. Have we not been inclined to overdo in the retention of pulpless teeth and roots in the jaws and were not the medical profession justified in their criticisms because of the indiscriminate saving of pulpless teeth? Dentists are more judicious in the matter and physicians consult with them more than formerly. I want to compliment the specialists of our city. They are more careful in diagnosis and invite consulation oftener. As for Dr. Sexton, he does not know much about dentistry.

Dr. J. Taft: Propably I can explain why Dr. Sexton takes such an interest in the teeth. He had two uncles who were dentists and he at one time thought of studying dentistry, and did study for a short time.

Often the inflammatory process extends from the molars to the tonsils and the throat.

Dr. H. A. Smith: Is it caused by septic matter carried from one point to another or is it nervous reflex action? Where we have a diseased condition of the alimentary tract, we have it manifested or reflected at the margin of the gums in pyorrhoea alveolaris.

Dr. J. Taft: You may call to mind many instances where the irritation is at one point and the manifestation far away.

Dr. C. M. Wright: The science of medicine and dentistry is not an exact one. We treat diseases empirically. The medical journals show that the treatment of diseases known for centuries varies from day to day. I know of instances where oculists have ordered out this or that tooth or of an amalgam filling ordered out for a special purpose. Instead they should send them to us for consultation, and not presume to dictate to us the course we should pursue.

TREATING AND FILLING DECIDUOUS TEETH.

There being no paper, the subject was opened by:

Dr. J. Taft: Prophylaxis is more important than filling. Induce a proper care and use of the teeth. Always arrest decay in its incipiency by cutting away, dressing down, or by filling in the best way possible. Cutting away is permissable to a greater extent than in the permanent teeth. The cements, tin, and gutta-percha are all good for filling. Many good operators use amalgam and I do not see that there is any special objection to its use in that way.

Dr. C. H. Harroun: One reason we know so little on this subject is that we have so few opportunities to do any thing. Another difficulty is that people will insist on having the deciduous teeth extracted and if you will not do it they will go elsewhere and have it done. As a filling I sometimes use tin and gold cylinders burnished in, or soft gold—in fact, any thing that will do.

Dr. Geo. W. Keely: The temporary teeth are as valuable to the child for masticating purposes as are the permanent ones to the adult. If you get the confidence of your little patients you can do almost any thing. I fill for children from two-and-a-half-years on. Whenever a temporary molar is extracted prematurely it will, in every case, cause a crowded condition of the permanent teeth, also, the early irruption of the permanent molars. The permanent first molar will in every instance come forward and occupy part of the room due the bicuspids. If there is decay in the temporary molar, proximal to the permanent one, cut away the decay with a disk.

Dr. H. A. Smith: The interdependence between the two sets of teeth has been questioned. That the regularity or normality of the second set depends upon the retention of the first. Charles Jones in a late work calls attention to the fact that where the entire temporary set had been lost a very regular and beautiful set of permanent teeth were errupted. It is doubtful if the stimulation of the deciduous teeth in mastication effects the permanent ones. The prime consideration should be the care of the teeth by parents. A child two-and-a-half years old was brought to me; the mother had cleaned its teeth two or three times a day and the teeth were absolutely perfect. The parents of the child have imperfect teeth ; therefore, I think it was the care bestowed upon the child's teeth rather than heredity. The roots of pulpless teeth are absorbed if not surrounded or bathed in pus. It is too late a day to discuss whether or not dead tissues are absorbed. There are thousands of sets of irregular teeth where all of the temporary teeth were retained. We do not know as much about these matters as we should because we do not make records, each day, of our observations.

Dr. C. H. Harroun: I think improper, or rather unphysiological marriages have much to do with the causes of irregularity of the teeth.

Dr. F. H. Rehwinkel: Each case must be judged by itself and not by rigid rules, only being guided by general rules to save going wrong. Laws cannot be laid down that will do for every case. Heredity and a mixing of races has more to do with irregularity than any thing else. (He related the case of a child's teeth with exposed pulps where he had to extract the teeth).

Dr. Geo. W. Keely: In case of exposed pulps of the deciduous teeth I use a little nitrate of silver dissolved over the pulp to deaden it. May have to repeat it next day.

Dr. J. Taft: Think the pulps, in the case cited by Dr. Rehwinkel, might have been removed without greater disturbance than that caused by the removal of the teeth.

Dr. A. W. Harlan: A masticating apparatus for a growing child is just as necessary as for a man, and it is unfortunate that we cannot do much for them because of a lack of knowledge by parents as to the necessity for their retention. I do not know that I can censure either the parents or the family physician. If parents would take in hand the care of these teeth, the general health of the child would be greatly improved. It is fortunate if they do not fall into the hands of a man who considers his time too valuable to be wasted in cultivating the management of the child. I have been especially careful to retain the *temporary cuspids* and *second molars* until they are "exuviated" as Dr. Patrick says. There is not as great danger to the regularity of the permanent set when the temporary first molar is lost as the second molar and cuspid. In spite of the theory that if all the temporary teeth were removed there would be no greater liability to irregularity of the permanent teeth, forces may be, and are, at work that tip forward the permanent first molar and close up that space. If we can retain the temporary first molar until the ninth year, and the second, until the tenth or eleventh year, depending on the locality, we will assure to a great extent the regularity of the permanant teeth. How accomplish this? By the exercise of a great deal of patience and the judicious filling of the teeth. Each tooth must be judged when it presents itself and be treated accordingly. When there are proximate decays and the pulps dead, the phosphates do not commend themselves for filling. You will get better results from the gutta-perchas than from amalgams, or cements, or tin and gold, or tin alone. Base-slate or red gutta-percha is more easily replaced and does not wear away as rapidly as Hill's Stopping or other gutta-perchas having mineral constituents. I often bridge the space between two molars with gutta-percha and have good results. If you leave the space food lodges there and in picking it out fillings are dislodged, so I prefer to bridge it. Have never bridged with amalgam. I bridge over only in certain cases; not all cases mind you, and only in living teeth. I never bridge over when the pulps are dead. The roots of pulpless teeth are absorbed or dissolved in a different manner from those of living teeth. If they do not come away when it is time for the irruption of the permanent teeth I extract them if I know them to be pulpless.

### DR. ROBINSON'S PAPER ON NEAR APPROACH TO AND DEVITALIZATION OF THE DENTAL PULP.

Dr. D. R. Jennings: I find that the men who save these exposed pulps do so in their minds. I mean those with pulps actually exposed and not those cases covered with a thin layer of decayed dentine where you would expose a pulp if you cut down a little more. As for the non-conductivity of the various phosphate cements, I had some of them tested by the well-known electrician Mr. Charles Bush, of Cleveland, and the result was:—Dawson's 7 per cent. less than gold; Fletcher's Porcelain 5 per cent. less, and the Ft. Wayne, or the non-secret, 13 per cent. less. People fifty or more years old have no dental pulps—they have mere filaments which are often very sensitive, but no pulps.

Dr. H. A. Smith: A simple way to test the relative conductivity of different filling materials, is to form uniform cylinders, on one end put wax and stand then on the other end on a lid over a pot of hot water. The melting of the wax will give you the facts in a general way, but not the percentage.

Dr. F. H. Rehwinkel: The application of chloride of zinc is the assassination of the pulp. When I meet a case that is hopeless I kill the pulp at once, use Squibb's arsenious acid without morphine or any thing else.

Dr. D. R. Jennings: When do you consider a case hopeless?

Dr. Rehwinkel: If the patient is healthy I always give the pulp a chance. If there is inflammation of the pulp I doubt if it ever recovers its normal condition. Climate and changes of weather have much to do with the success or failure of the treatment. Malaria in the system impairs nutrition to such an extent that operations in a malarious district fail, whereas they would be successful in another part of the country.

(Dr. Betty's Paper Tin and Gold Fillings.)

Dr. A. W. Harlan—Have used a great deal of the tin and gold combination in filling cavities entirely and partially. The specimens of the material as presented by Dr. Betty are not nearly so compact as I use them. Have never used them quite so loosely twisted together. For ropes, I cut the sheets into two pieces, and on rolling endeavor to have a fine streak of gold exposed and the remainder tin. In finishing a filling with gold I depend on forcing the gold into the mass of tin and gold with deeply serated instruments with wooden handles. In a proximal cavity in a bicuspid with live pulp, one rope will fill one-third of the cavity. Am not particular about condensing the tin and gold when placing it in the cavity. I put a piece in one corner, another in the other and one between the two. It is wonderful how well they remain where you put them. If using a matrix I feel a security, against a recurrence of decay, in using tin and gold that I do not with cohesive gold. It does not seem to require the same amount of force as gold to impact it against the walls. Tin and gold in blackening does not seem to penetrate the dentine nor discolor the surface. It is non-shrinkable. It doesn't rock in the cavity. It does not require such a loss of tissue in the preparation of the cavity as for gold. It allows of making retaining groves in such part of the tooth as to least endanger the pulp. It is far superior to all amalgams except the precipitated copper or palladium amalgams, the method of preparing which will be made public in a few weeks. Dr. George H. Weagant, of Cornwall, Ontario, claims to have invented a copper amalgam that will not stain the tooth. [See the DENTAL REVIEW, November, 1887.] Tin and gold keeps the ordinary dentist up to his mark. He must know how to use it, or the fillings will disintegrate. There are many people who cannot afford to pay for gold or some teeth that are not strong enough to stand the gold. Another advantage is that tin and gold may be put in under moisture or without the use of the dam.

Dr. J. R. Callahan: I use the Herbst method in filling with tin and gold. Can make a harder filling than by mallet pressure. Use a four ounce mallet.

Dr. E. G. Betty: Try a twelve ounce mallet.

Dr. Callahan: Don't want to after having had you try one on me.

Dr. W. H. Sedgwick: Always have the sheet of tin smaller than the gold so as to have the gold on the outside of the rope. I got the method of using it from Dr. Miller and it has proven very satisfactory.

Dr. E. G. Betty: I finish with No. 60, gold foil, annealing it and driving it into the mass of tin and gold. A tin and gold filling is far more to the credit of the operator than sticking a lump of amalgam into a cavity with his thumb. Dr. Harlan thinks the therapeutic effect is from a sulphide. If it is a sulphide, it is possible the sulphide is formed thus:—Tin and gold in contact form the plates of a battery both being equally exposed to the fluids of the mouth. Albumen, which is present in the mouth, contains sulphur; the decomposition of this by electrolysis would liberate sulphur, which might immediately unite with the tin to form the sulphide. I am inclined to think, however, that it is $SO^3$, the acid radical of sulphuric acid, and that it is a combination of this with the tin which takes place, the tin taking the place of the oxygen. I formerly used a great deal of Robinson's fibrous foil, but find the tin and gold easier to use and adapt to the cavity. On removing old fillings of the former I find them completely disintegrated, or powdery.

Dr. J. Taft: Two have used the term "amalgam without mercury." If I am not mistaken the defination of an amalgam is a combination of metals, or an alloy, with mercury.

Dr. C. M. Wright: We can use the term if we wish. I think Prof. Taft will admit that if a black man marries a white woman, there is an amalgamation of the race without mercury, unless the man has been taking calomel. We used tin and gold in Europe about fifteen years ago, rolled until it looked like a barber's pole.

Dr. P. G. C. Hunt: Has any one observed any difference between the action of tin alone and of the tin and gold on a tooth structure in the cavity? If there is anything better than pure tin for filling cavities in soft teeth I want to use it.

Dr. H. A. Smith: If we put a filling of gold into a cavity of sensitive dentine, we induce a low grade of irritation that invites or causes a flow of lime salts to the parts and a consolidation of the dentine under the filling. With tin and gold we have less conductivity and less irritation. Now does this allow of or induce the consolidation of the dentine under it, or which one results in the greatest solidification.

Dr. J. Taft: Under the gold this solidification cannot take place, because of the rapid transmission of thermal changes, while the non-conductivity of the tin and gold allows of it. I am not wholly satisfied with this admixture of metals, but think it worthy of experiment. When an old filling of tin and gold is broken up it is found to be granular rather than crystalline, which accounts for the density of the filling.

